# Joint Bodies from Within and Without, Present in Articulations Otherwise Apparently Normal

**Published:** 1915-02

**Authors:** 


**Joint Bodies From Within and Without, Present in Articulations Otherwise Apparently Normal.** Aime Paul Heineck, "Ill. Med. Jour.," Jan., 1915. Not amenable to abstracting but one of the most thorough statistic studies published. Those interested are advised to consult the original.

